# Risk factors for delayed viral suppression on first-line antiretroviral therapy among persons living with HIV in Haiti, 2013–2017

**DOI:** 10.1371/journal.pone.0240817

**Published:** 2020-10-29

**Authors:** Shannan N. Rich, Robert L. Cook, Lusine Yaghjyan, Kesner Francois, Nancy Puttkammer, Ermane Robin, Jungjun Bae, Nadjy Joseph, Luisa Pessoa-Brandão, Chris Delcher

**Affiliations:** 1 Department of Epidemiology, College of Public Health and Health Professions and College of Medicine, University of Florida, Gainesville, FL, United States of America; 2 Department of Health Outcomes and Biomedical Informatics, College of Medicine, University of Florida, Gainesville, FL, United States of America; 3 Programme National de Lutte contre le SIDA, Ministère de la Santé Publique et de la Population, Port au Prince, Haïti; 4 Department of Global Health, International Training and Education Center for Health (I-TECH), University of Washington, Seattle, WA, United States of America; 5 College of Pharmacy, Institute for Pharmaceutical Outcomes and Policy, University of Kentucky, Lexington, KY, United States of America; 6 National Alliance of State and Territorial AIDS Directors, Port au Prince, Haiti; 7 Department of Pharmacy Practice and Science, College of Pharmacy, University of Kentucky, Lexington, KY, United States of America; National Institute for Communicable Disease (NICD), South Africa, SOUTH AFRICA

## Abstract

Studies of viral suppression on first-line antiretroviral therapy (ART) in persons living with human immunodeficiency virus (PLHIV) in Haiti are limited, particularly among PLHIV outside of the Ouest department, where the capital Port-au-Prince is located. This study described the prevalence and risk factors for delayed viral suppression among PLHIV in all geographic departments of Haiti between 2013 and 2017. Individuals who received viral load testing 3 to 12 months after ART initiation were included. Data on demographics and clinical care were obtained from the Haitian Active Longitudinal Tracking of HIV database. Multivariable logistic regression was performed to predict delayed viral suppression, defined as a viral load ≥1000 HIV-1 RNA copies/mL after at least 3 months on ART. Viral load test results were available for 3,368 PLHIV newly-initiated on ART. Prevalence of delayed viral suppression was 40%, which is slightly higher than previous estimates in Haiti. In the multivariable analysis, delayed viral suppression was significantly associated with younger age, receiving of care in the Ouest department, treatment with lamivudine (3TC), zidovudine (AZT), and nevirapine (NVP) combined ART regimen, and CD4 counts below 200 cells/mm^3^. In conclusion, this study was the first to describe and compare differences in delayed viral suppression among PLHIV by geographic department in Haiti. We identified populations to whom public health interventions, such as more frequent viral load testing, drug resistance testing, and ART adherence counseling should be targeted.

## Introduction

Careful adherence to effective antiretroviral therapy (ART) for people living with human immunodeficiency virus (PLHIV) leads to suppression of plasma viral loads to undetectable levels.

Delays in viral suppression can lead to virologic failure, formally defined by the World Health Organization (WHO) in developing countries as the failure to achieve a plasma viral load level below 1,000 HIV-1 RNA copies/mL after three months on ART [1]. Virologic failure is associated with higher rates of mortality, immunosuppression and the accumulation of resistance mutations [2, 3] and individuals who are not suppressed have a much higher probability of transmitting the virus to others [3].

Haiti has the highest adult HIV prevalence in the Western hemisphere [4]. Despite this, few studies have characterized the viral suppression status of PLHIV in this population [5]. Viral load assessments were introduced in five HIV care facilities in Haiti as part of a pilot program in 2013 and were officially scaled-up nationally in 2016 [6]. Previous studies conducted among clinics located in the capital of Port-au-Prince reported virologic failure prevalence estimates between 32% and 51% [6, 7]. Factors associated with virologic failure in one study included male sex, lower CD4 (<500 cells/mm^3^), poor adherence to ART, and tuberculosis co-infection [6]. Major drug regimen changes–an indicator of failing drug therapies suggestive of virologic failure–have also been assessed in Haiti, with another study reporting positive associations with female sex, younger adult age (20–39 years old), ART initiation year after 2006, and lower CD4 (<350 cells/mm^3^) at ART initiation [8]. To date, few studies have assessed the virologic outcomes of PLHIV newly-initiated on ART or who reside outside of Port-au-Prince. The aims of this study were to 1) estimate the prevalence of delayed viral suppression among PLHIV recently initiated on ART in Haiti and 2) determine key demographic, geographic and clinical characteristics associated with delayed viral suppression. We hypothesized that the likelihood of delayed viral suppression varies according to demographic and clinical risk and protective factors informed by the socio-ecologic model [9].

## Methods

### Study population

We employed a retrospective cohort design to assess the virologic outcomes of treatment naïve PLHIV individuals newly-initiated on ART in Haiti. Data were accessed using the national HIV case-based surveillance system, the Haitian Active Longitudinal Tracking of HIV database (SALVH, French acronym), which has been operated by the Ministry of Public Health and Population (MSPP) since 2008 and previously described elsewhere [5, 10]. Briefly, there are three main health networks that provide treatment services to PLHIV in Haiti and report electronic medical records (EMR) to SALVH, including the MSPP-affiliated clinics using the iSanté EMR network (administered by the International Training and Education Center for Health), the Haitian Group for the Study of Kaposi’s Sarcoma and Opportunistic Infections (GHESKIO), and Partners in Health’s Zanmi Lasante (PIH) [10]. For this analysis, we included all PLHIV who received their first ART prescription between January 1, 2013 and December 31, 2016 and who had a viral load test performed between 3–12 months (90–365 days) after ART initiation recorded in SALVH. Laboratory data were collected until December 31, 2017 to permit a full year of follow-up for all participants.

### Ethics approval

This study was reviewed by, and received ethics approval from, the National Bioethics Committee in Haiti (reference: 1718–37) and the Institutional Review Board at the University of Florida (reference: IRB201702830). We received a full waiver of informed consent and the study was approved as expedited since the data were collected originally for non-research purposes.

### Delayed viral suppression

In Haiti’s pilot phase of viral load testing roll-out (July 2013 –October 2016), plasma blood samples were collected at clinic facilities and transported to the National Public Health Laboratory (PNLS, French acronym) in Port-au-Prince where viral load levels were accessed using the Generic HIV Viral Load Assay (Biocentric, Bandol, France). This test has a limit of detection of 300 copies/mL and detailed methods regarding its use in this population have previously been described elsewhere [6]. Starting after October 2016, dried blood spot (DBS) analyses (limit of detection = 550–1000 copies/mL) [11] were introduced using the Abbott Real-Time HIV-1 assay. The performance of DBS analysis for viral load monitoring is comparable to plasma viral load monitoring and is recommended in low resource environments where capacity for plasma storage and sample processing is limited [11, 12]. Briefly, 75ul of whole blood was collected from each patient by a trained lab technician at each facility. Five blood spots were applied to DBS filter paper strips and dried at room temperature for four hours on a drying rack. The DBS samples were stored individually in a ziplocked envelope containing desiccants and a humidity indicator card. Envelopes were transported twice weekly to PNLS for viral load assessment. PLHIV with a viral load test result ≥1,000 HIV-1 RNA copies/mm^3^ between 3 to 12 months after ART initiation were classified as having delayed viral suppression. PLHIV whose viral load test result was <1,000 copies/mm^3^ were classified as virally suppressed. In the event a patient had more than one viral load test performed in the 3-12-month window after ART initiation, we selected the earliest test result for the analysis.

### Predictors

Candidate risk and protective factors were accessed at the time of the initial HIV case report and included age, sex, marital status, and geographic department of clinic (Haiti is administratively organized into 10 geographic departments). An individual’s clinical characteristics included dates of HIV diagnosis and ART initiation, time from diagnosis to ART initiation (termed “time to ART”), earliest recorded CD4 T-cell count (cells/mm^3^), ART drug regimen (combinations of three possible drugs, including one non-nucleoside reverse transcriptase (NNRTI): nevirapine [NVP] or efavirenz [EFV] and two nucleoside-reverse transcriptase inhibitors [NRTIs]: lamivudine [3TC], zidovudine [AZT], or tenofovir [TDF]; or “other” if the regimen comprised less than three medications or contained other less-frequently prescribed medications), WHO clinical stage of HIV disease (scale of 1 to 4, where 4 is the most severe) [13], transmission risk factors identified on the HIV case-report form, history or presence of tuberculosis (TB) or sexually transmitted infections (STIs), and whether individuals received ART from one or multiple clinics during the study period (termed “multiple treatment facilities”). Individuals with missing data on age, sex, clinic department, dates of diagnosis and ART initiation, or ART regimen were excluded from the inferential analyses. Large numbers of missing values in other covariates were categorized as “unknown” or “missing” and retained for the regression analysis.

### Statistical analysis

First, we described the frequency of viral load testing and frequency of delayed viral suppression among those tested. Demographic and clinical characteristics of individuals with and without delayed viral suppression were compared using the chi-squared test for categorical variables and two sample t-tests for age as a continuous variable. Variables that were significantly different (p<0.05) by virologic status were examined in bivariate analyses and then included in the logistic regression analysis. The full model included: age (years), sex (male or female [reference]), marital status (cohabiting, married, or divorced/widowed, single [reference]), and clinic department of location (Ouest [reference], Artibonite-Centre, Nord-Est, Nord-Ouest, Sud [includes Sud, Sud-Est, Grande-Anse, and Nippes], or Nord) as well as clinical factors: year of HIV diagnosis (<2010 [reference], 2010–11, 2012–13, 2014–15, 2016), WHO clinical stage (I [reference], II, III, or IV), year of ART initiation (2013 [reference], 2014, 2015, or 2016), first-line ART regimen (3TC-EFV-TDF [reference], 3TC-AZT-NVP, 3TC-EFV-AZT, or other), earliest CD4 count (<200, 200–499, or 500+ cells/mm^3^ [reference]), having one or more HIV transmission risk factor identified on the case report (yes, no [reference], or unknown), and receipt of ART from multiple treatment facilities (yes, no [reference], or unknown). A parsimonious model was also fitted using a stepwise backwards-elimination variable selection procedure (based on Akaike’s information criterion); however, the results of the parsimonious model were similar to the results of the full model (i.e., all variables were retained), and therefore only the full model results were presented. To examine the effect of geographic residence, we ran the full models with and without department of residence and the findings remained the same.

The associations were described as odds ratios (OR) and their respective 95% confidence intervals (CI) at the p<0.05 significance level. SAS software version 9.4 was used for all data analyses [14]. We plotted the prevalence of delayed viral suppression by geographic department using the Geographic Information Systems (GIS) software, ArcGIS Pro 2.3.3 [15].

## Results

Between 2013 and 2017, 6,395 PLHIV had viral load tests documented in SALVH within 3–12 months after ART initiation; however, the results of the tests were not available for a substantial number of individuals (n = 3,027), presumably due to reporting issues. We assessed whether there were any systematic differences between the characteristics of PLHIV who had test results reported to the SALVH surveillance system (n = 3,368) vs those who did not have reliable test results reported to SALVH (n = 3,027) (refer to S1 Table). Of note, individuals included in the study population were more likely to be treated with 3TC-EFV-TDF regimens (87.9% vs 84.5%, p<0.01) and have a CD4 count <200 cells/mm^3^ (24.0% vs 15.1%, p<0.01).

### Characteristics of study population

**Fig 1:**
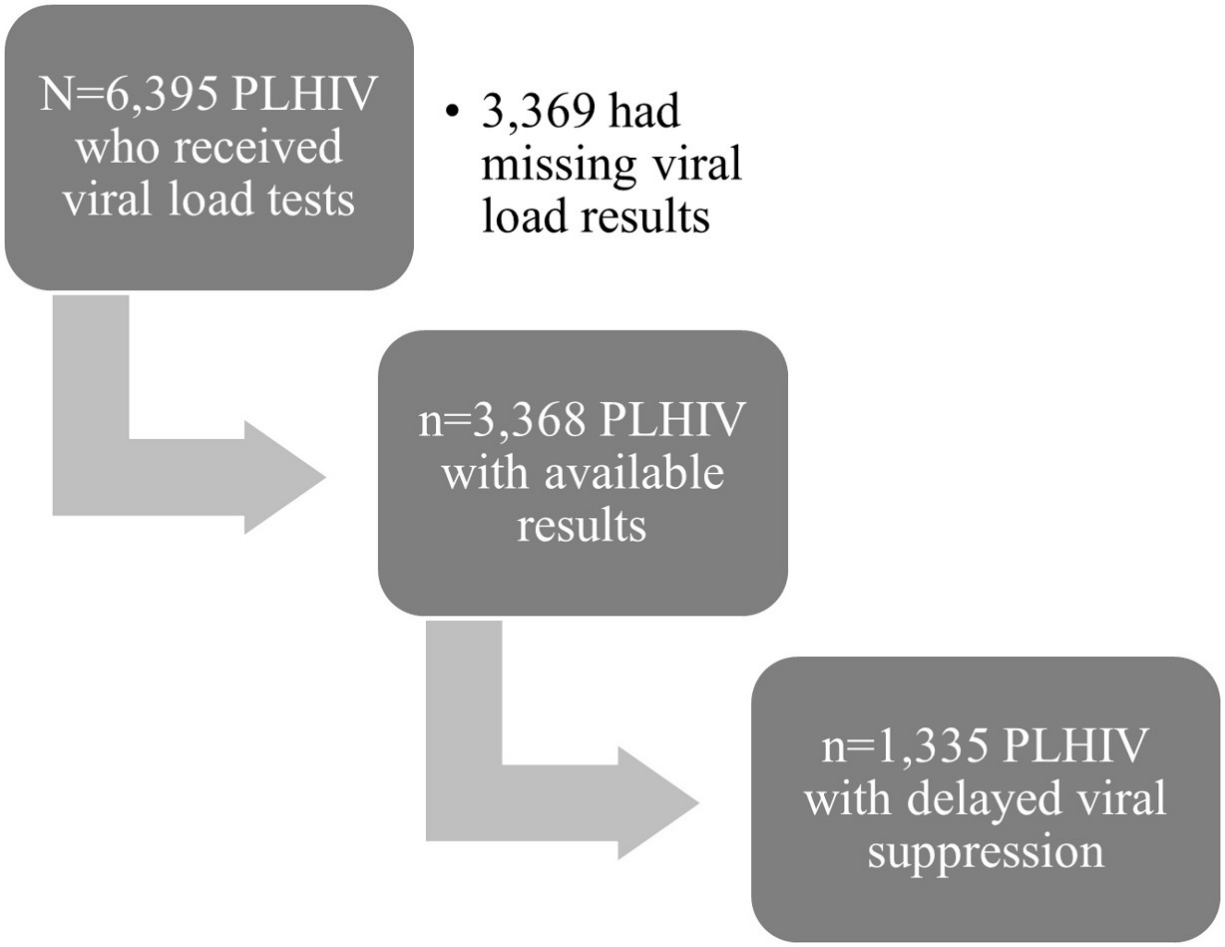
Selection of the study population. This figure displays the participation disposition flow for the study.

**Fig 2:**
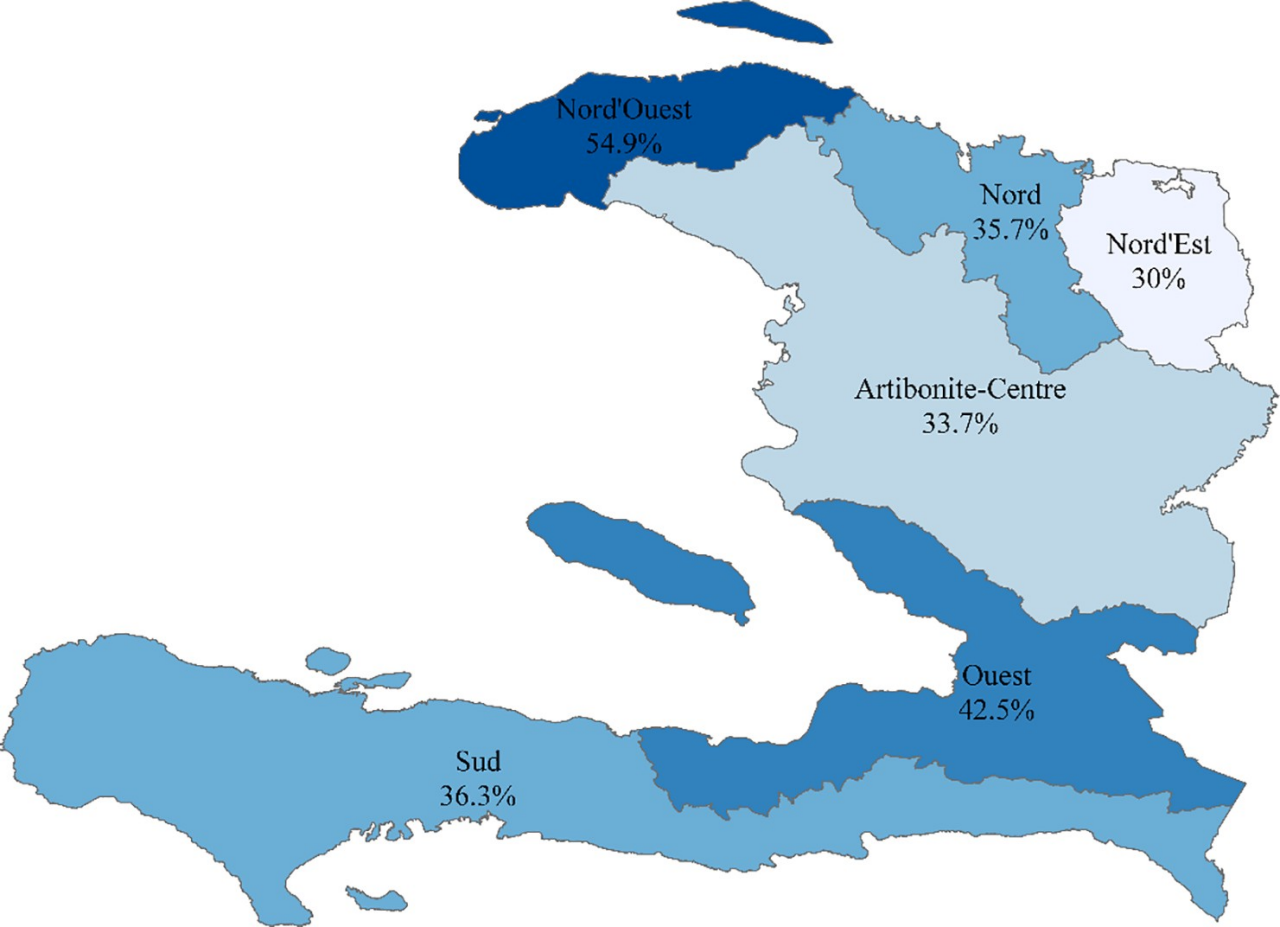
Prevalence of delayed viral suppression by geographic region (department) in Haiti between 2013 and 2017. This figure shows the prevalence of delayed viral suppression among persons newly-initiated on antiretroviral therapy by the geographic department in which they received clinical care between 2013 and 2017. ‘Sud’ department in this image represents Grande Anse, Nippes, Sud, and Sud-Est departments combined.

Among PLHIV with available viral load test results, 1,335 had a result ≥1,000 HIV-1 RNA copies/mm^3^, indicating a prevalence of delayed viral suppression of 39.6% in this sample. A flowchart showing the selection of the population is depicted in Fig 1. The median time to viral load measurement was 216 days for those included in the study. The average log-transformed viral load values were consistent across all months of assessment after ART initiation (S1 Fig). Prevalence of delayed viral suppression varied across geographic departments: 54.9% in the Nord-Ouest, 35.7% in the Nord, 30% in the Nord-Est, 33.7% in Artibonite-Centre, 42.5% in the Ouest, 36.3% in the Sud (Fig 2).

Descriptive analyses indicated that individuals who experienced delayed viral suppression were on average younger than individuals with suppression (33.2 vs 36.3 years, p<0.01) and more likely to be under the age of 20 years (11.7% vs 6.8%, p<0.01) when examining age groups (Table 1). Those experiencing delayed virologic suppression were more likely to be male (43.1% vs 39.5%, p = 0.04), diagnosed in the Ouest Department (61.1% vs 54.3%, p<0.01), have a CD4 count test result <200 cells/mm^3^ (29.0% vs 20.8%, p<0.01), and to receive ART from multiple treatment facilities (5.9% vs 3.6%, p<0.01), as compared to individuals with virologic suppression. PLHIV who experienced delayed viral suppression were also less likely to be married (12.4% vs 15.5%, p<0.01) or have an HIV transmission risk factor identified on a case-report form (51.5% vs 55.6%, p = 0.02). The majority of PLHIV in both populations were prescribed 3TC-EFV-TDF regimens, however individuals with delayed viral suppression were more likely to receive an ART regimen other than 3TC-EFV-TDF (16.3% vs 9.4%, p<0.01). PLHIV with and without delayed viral suppression did not appear to differ with respect to time from diagnosis to ART initiation or history or presence of TB or STIs.

**Table 1:** Demographic and clinical characteristics of persons living with HIV in Haiti by virologic status, 2013–2017.

Characteristic	Delayed viral suppression (n = 1,335)	Viral suppression (n = 2,033)		Global p-for difference
				χ^2^ test
Mean (SD)				
Age at HIV diagnosis (years)*	33.2 (14.0)	36.3 (13.0)		<0.0001
Frequency (%)				
Age at HIV diagnosis (years)				<0.0001
<5	65 (4.9)	33 (1.6)		
5–14	47 (3.5)	48 (2.4)		
15–19	44 (3.3)	57 (2.8)		
20–29	343 (25.7)	470 (23.1)		
30–39	400 (30.0)	634 (31.2)		
40–49	247 (18.5)	440 (21.6)		
50–59	119 (8.9)	231 (11.4)		
60+	37 (2.8)	82 (4.0)		
Unknown	33 (2.5)	38 (1.9)		
Sex				0.0352
Female	759 (56.9)	1,230 (60.5)		
Male	576 (43.1)	803 (39.5)		
Marital status				0.0012
Single	278 (20.8)	374 (18.4)		
Cohabitating	516 (38.7)	778 (38.3)		
Married	166 (12.4)	315 (15.5)		
Divorced or widowed	151 (11.3)	288 (14.2)		
Unknown	224 (16.8)	278 (13.7)		
Clinic department^†^				<0.0001
Artibonite-Centre	94 (7.0)	185 (9.1)		
Nord	229 (17.2)	413 (20.3)		
Nord-Est	48 (3.6)	112 (5.5)		
Nord-Ouest	45 (3.4)	37 (1.8)		
Ouest	816 (61.1)	1,103 (54.3)		
Sud	101 (7.6)	177 (8.7)		
Unknown	2 (0.1)	6 (0.3)		
Year of HIV diagnosis			0.0052	
<2010	78 (5.8)	117 (5.8)		
2010–11	84 (6.3)	116 (5.7)		
2012–13	204 (15.3)	314 (15.4)		
2014–15	535 (40.1)	939 (46.2)		
2016	430 (32.2)	539 (26.5)		
Unknown	4 (0.3)	8 (0.4)		
WHO clinical stage			<0.0001	
I	163 (12.2)	223 (11.0)		
II	369 (27.6)	440 (21.6)		
III	165 (12.4)	199 (9.8)		
IV	29 (2.2)	29 (1.4)		
Unknown	609 (45.6)	1,142 (56.2)		
Year of ART initiation			0.0225	
2013	107 (8.0)	123 (6.1)		
2014	108 (8.1)	153 (7.5)		
2015	374 (28.0)	652 (32.1)		
2016	746 (55.9)	1,105 (54.4)		
ART regimen			<0.0001	
3TC-AZT-NVP	79 (5.9)	54 (2.7)		
3TC-EFV-AZT	57 (4.3)	73 (3.6)		
3TC-EFV-TDF	1,118 (83.7)	1,841 (90.6)		
Other	81 (6.0)	65 (3.0)		
Earliest CD4 T cell count			<0.0001	
<200	387 (29.0)	423 (20.8)		
200–499	389 (29.1)	660 (32.5)		
500+	274 (20.5)	566 (27.8)		
Unknown	285 (21.3)	384 (18.9)		
History/presence of TB			0.0828	
No	803 (60.1)	1,299 (63.9)		
Yes	62 (4.6)	91 (4.5)		
Unknown	470 (35.2)	643 (31.6)		
History/presence of STIs			0.5356	
No	663 (49.7)	990 (48.7)		
Yes	288 (21.6)	472 (23.2)		
Unknown	384 (28.8)	571 (28.1)		
Any risk factor identified			0.0196	
No	647 (48.5)	902 (44.4)		
Yes	688 (51.5)	1,131 (55.6)		
Multiple ART facilities			0.0019	
No	1,256 (94.1)	1,959 (96.4)		
Yes	79 (5.9)	74 (3.6)		
Time to ART			0.0013	
<1 year of diagnosis	945 (70.8)	1,409 (69.3)		
≥1 year of diagnosis	353 (26.4)	599 (29.5)		
Unknown	37 (2.8)	25 (1.2)		

SD, standard deviation; WHO, World Health Organization; ART, antiretroviral therapy; 3TC, lamivudine; AZT, zidovudine; NVP, nevirapine; EFV, efavirenz; TDF, tenofovir; TB, tuberculosis; STIs, sexually transmitted infections.

*Evaluated using t-test.

†Sud department is combined and includes Sud, Sud-Est, Grande-Anse, Nippes.

### Associations of demographic and clinical characteristic with delayed viral suppression

Bivariate and multivariate results are presented in Table 2. In the multivariable analysis, age, clinic department, ART regimen, and CD4 count remained significantly associated with delayed viral suppression at the p<0.05 level. Age was inversely correlated with delayed viral suppression, for which we observed a 1% decrease in the likelihood of delayed suppression for each one-year increase in age (OR = 0.99; 95% CI 0.98–0.99) (Table 2). PLHIV diagnosed in the Artibonite-Centre, Nord, and Nord-Est departments had 26%, 20%, and 39% decreased odds of delayed suppression, respectively, as compared to PLHIV diagnosed in the Ouest department. The demographic characteristics of sex and marital status were not significantly associated with viral suppression status in the multivariable models.

**Table 2:** Odds Ratios (OR) and 95% Confidence Intervals (CI) of demographic and clinical characteristics associated with delayed viral suppression in Haiti, 2013–2017.

Characteristic	Unadjusted ORs	Adjusted ORs
	(95% CI)	(95% CI)
Age at HIV diagnosis (years)	0.98 (0.98; 0.99)	0.99 (0.98; 0.99)
Sex		
Male *vs*. *female*	1.16 (1.01;1.34)	1.14 (0.98; 1.33)
Marital status		
Cohabitating *vs*. *single*	0.89 (0.74; 1.08)	1.10 (0.90; 1.36)
Married *vs*. *single*	0.71 (0.56; 0.91)	0.87 (0.66; 1.13)
Divorced/widowed *vs*. *single*	0.71 (0.55; 0.91)	0.91 (0.68; 1.21)
Unknown *vs*. *single*	1.08 (0.86; 1.37)	1.13 (0.88; 1.46)
Clinic department*		
Artibonite-Centre *vs*. *Ouest*	0.69 (0.53; 0.90)	0.74 (0.56; 0.99)
Nord *vs*. *Ouest*	0.75 (0.62; 0.90)	0.80 (0.64; 0.99)
Nord-Est *vs*. *Ouest*	0.58 (0.41; 0.82)	0.61 (0.42; 0.89)
Nord-Ouest *vs*. *Ouest*	1.64 (1.05; 2.56)	1.45 (0.90; 2.35)
Sud *vs*. *Ouest*	0.77 (0.59; 1.00)	0.84 (0.63; 1.12)
Unknown *vs*. *Ouest*	0.45 (0.09; 2.24)	0.45 (0.09; 2.32)
Year of HIV diagnosis		
2010–11 *vs*. *<2010*	1.09 (0.73; 1.62)	1.22 (0.79; 1.86)
2012–13 *vs*. *<2010*	0.98 (0.70; 1.36)	1.02 (0.69; 1.49)
2014–15 *vs*. *<2010*	0.86 (0.63; 1.16)	0.95 (0.63; 1.42)
2016 *vs*. *<2010*	1.20 (0.88; 1.64)	1.23 (0.76; 2.01)
Unknown *vs*. *<2010*	0.75 (0.22; 2.58)	-^†^
WHO clinical stage		
II *vs*. *I*	1.15 (0.90; 1.47)	1.20 (0.93; 1.57)
III *vs*. *I*	1.13 (0.85; 1.51)	1.10 (0.81; 1.51)
IV *vs*. *I*	1.37 (0.79; 2.38)	1.48 (0.82; 2.67)
Unknown *vs I*	0.73 (0.58; 0.91)	0.80 (0.62; 1.04)
Year of ART initiation		
2014 *vs*. *2013*	0.81 (0.57; 1.16)	0.91 (0.58; 1.42)
2015 *vs*. *2013*	0.66 (0.49; 0.88)	0.90 (0.60; 1.35)
2016 *vs*. *2013*	0.78 (0.59; 1.02)	0.99 (0.64; 1.55)
ART regimen		
3TC-AZT-NVP *vs*. *3TC-EFV-TDF*	2.41 (1.69; 3.43)	2.18 (1.48; 3.23)
3TC-EFV-AZT *vs*. *3TC-EFV-TDF*	1.29 (0.90; 1.83)	1.41 (0.96; 2.07)
Other *vs*. *3TC-EFV-TDF*	2.14 (1.52; 3.02)	1.66 (1.14; 2.43)
Earliest CD4 T cell count		
<200 *vs*. *500+*	1.89 (1.55; 2.31)	1.90 (1.51; 2.39)
200–499 *vs*. *500+*	1.22 (1.01; 1.47)	1.30 (1.05; 1.61)
Unknown *vs*. *500+*	1.53 (1.24; 1.89)	1.64 (1.30; 2.07)
Any risk factor identified		
Yes *vs*. *no*	0.85 (0.74; 0.97)	0.96 (0.83; 1.12)
Multiple treatment facilities		
Yes *vs*. *no*	1.67 (1.20; 2.30)	1.28 (0.88; 1.85)
Time to ART		
≥ 1 year of diagnosis *vs*. *<1 year of diagnosis*	0.88 (0.75; 1.03)	0.94 (0.69; 1.28)
Unknown *vs*. *<1 year of diagnosis*	2.21 (1.32; 3.69)	2.02 (1.04; 3.93)

OR, odds ratio; CI, confidence interval; WHO, World Health Organization; ART, antiretroviral therapy; 3TC, lamivudine; AZT, zidovudine; NVP, nevirapine; EFV, efavirenz; TDF, tenofovir.

*Sud department is combined and includes Sud, Sud-Est, Grande-Anse, Nippes.

†Missing values were dropped due to removal of missing values in another data field.

Individuals treated with 3TC-AZT-NVP or an alternative (“other”) regimen had approximately 2.2 (OR = 2.18; 95% CI 1.48–3.23) and 1.7 (OR = 1.66; 95% CI 1.14–2.43) times increased odds of delayed viral suppression, respectively, as compared to individuals treated with 3TC-EFV-TDF. Compared to individuals in the highest CD4 category (≥500 cells/mm^3^), individuals in the lowest category (<200 cells/mm^3^) had 1.9 times increased odds of delayed viral suppression (OR = 1.90; 95% CI 1.51–2.39). Individuals in the second lowest CD4 category (200–499 cells/mm^3^) had 1.3 times increased odds of delayed viral suppression (OR = 1.31; 95% CI 1.06–1.61) compared to individuals in the highest CD4 category. Interestingly, individuals with missing CD4 values performed similarly to those within the lowest CD4 category compared to those within the highest category (OR = 1.92; 95% CI 1.53–2.40). Individuals with missing data on time to ART had higher odds of delayed viral suppression compared to individuals who initiated ART within one year of diagnosis (OR = 2.02; 95% CI 1.04–3.93). WHO clinical stage, years of HIV diagnosis or ART initiation, having a risk factor identified, and receipt of ART from multiple facilities were not statistically significantly associated with delayed viral suppression in the final multivariable models.

## Discussion

The purpose of this study was to estimate the prevalence of and determine the demographic and clinical characteristics associated with delayed viral suppression in a sample of PLHIV receiving first line ART in Haiti. The results indicated virologic outcomes varied according to age, clinic department, ART regimen, and CD4 count. Delayed viral suppression occurred in nearly 40% of this sample, which is slightly higher than current estimates of this outcome in Haiti. In the analysis conducted by Jean Louis et al., 32.4% of individuals experienced a viral load level ≥1,000 copies/mL after 6–60 months or more on ART [6]. Most individuals in that study (84%) were receiving ART for at least 12 months prior to viral load assessment [6], and the analysis did not account for regimen changes during the follow-up period, which could indicate delays in viral suppression or virologic failure. In considering the geographic distribution of viral suppression status, we observed the highest prevalence of delayed suppression among patients receiving care at clinics located in the Ouest (42.5%) and Nord-Ouest departments (54.9%); however, only the Ouest department remained significantly associated with increased odds of delayed suppression after multivariable adjustment.

The geographic variability identified in our analysis could perhaps be due to surveillance bias and regional differences in resources. Clinicians in the Ouest department, where the capital Port-au-Prince and the majority of HIV facilities are located, may have been more likely to order viral load tests for those suspected of virologic failure, allowing for a higher rate of detection than may be possible in regions with less resources. Although not statistically significant in the multivariable model, the prevalence of delayed viral suppression was highest in the Nord-Ouest department and this finding is similar to results presented in another study assessing rates of mortality and loss to follow-up after ART initiation by specific HIV facility in Haiti [4]. That study reported higher rates of mortality in the main HIV facility in the Nord-Ouest (9.5 deaths per 100 person years in the first year following ART initiation) versus the Ouest (1.6–8.3 deaths per 100 person years) [4]. Rates of loss to follow-up were not noticeably higher in the Nord Ouest facility, however, according to the same study.

In addition to receiving care in the Ouest department, younger patients were more likely to experience delayed viral suppression. Delays in viral suppression on first-line ART among younger PLHIV may be linked to issues of adherence or pretreatment drug resistance among persons with perinatal exposure to HIV in Haiti, where rates of mother-to-child transmission in the early years of the epidemic were reportedly as high as 27% [16]. According to the WHO’s recent report on HIV drug resistance, the prevalence of pretreatment resistance is disproportionally higher among children infected through mother-to-child-transmission [17]. In contrast with our findings, however, Jean Louis et al reported younger age was not associated with long-term viral suppression status [6]. There was no association between marital status and sex with viral suppression in this study after multivariable adjustment. The lack of a statistically significant difference in treatment outcomes between males and females stands in contrast to previous studies in Haiti. In Jean Louis et al., males were 20% less likely to be virally suppressed after at least 6 months on ART [6] and in McNairy et al., male sex was associated with higher HIV mortality after 12 months on ART [4]. While it is possible that males may have poorer health outcomes in the long term due to issues such as adherence or loss to follow-up [4, 6], our study found no difference in early virologic outcomes by sex after multivariable adjustment.

The clinical factors associated with increased odds of delayed viral suppression in our analysis were a CD4 count <200 cells/mm^3^, an ART regimen consisting of 3TC-AZT-NVP and receipt of ART from multiple treatment facilities. The association between delayed suppression and lower CD4 counts is consistent with previous studies in Haiti and in other low resource environments [6, 8, 18]. Missing CD4 counts appear to be an indicator of poorer health status given that they were associated with delayed viral suppression. While most individuals in our study were receiving the current recommended ART regimen (3TC-EFV-TDF), individuals on alternative regimens were more likely to experience delayed suppression. Notably, the ART regimen 3TC-AZT-NVP was linked to the greatest odds of delayed viral suppression in this study when compared to the 3TC-EFV-TDF regimen. This first-line ART regimen was similarly associated with delayed viral suppression in the study conducted by Jean Louis et al. Differences in virologic outcomes on 3TC-AZT-NVP vs 3TC-EFV-TDF regimens may be explained by the comparative effectiveness of the NRTI backbone [AZT vs TDF] or the NNRTIs [\NVP vs EFV]. Previous studies comparing the efficacy and effectiveness of TDF- vs AZT-based regimens have reported better performance of TDF with respect to higher immunologic recovery [19], fewer drug substitutions and lower mortality [20]. Moreover, a systematic review of the effectiveness of EFV versus NVP NNRTI therapies reported minimal difference in likelihood of viral suppression, however, the development of drug resistance was slightly higher for individuals who received NVP [21]. Poorer efficacy and effectiveness of AZT or NVP alone, or in combination, may explain the poorer virologic outcomes observed among persons receiving 3TC-AZT-NVP regimens in this study. Receipt of ART from multiple treatment facilities was positively correlated to delayed viral suppression in our study, suggesting that transfers between clinics could be a signal for medication shopping due to failed treatments or fragmented care.

This analysis was the first to assess the virologic outcomes of PLHIV outside of the capital of Port-au-Prince and to report variability by geographic regions (departments). Moreover, this analysis was also the first to assess delayed viral suppression among PLHIV newly-initiated on ART in Haiti, which provides important insight for informing national treatment guidelines for first-line ART. Previous studies have reported that earlier detection of unsuppressed viral load is highly correlated with long-term virologic failure [22, 23] and significantly reduces delays in switching to second-line therapies [24]. Earlier detection of delayed viral suppression and switch to second-line therapy was previously found to increase the probability of survival among PLHIV in Haiti from 88% to 93% [25].

This study had limitations. First, according to the surveillance system, viral load tests were performed for only 35.8% of PLHIV newly-initiated on ART between 2013 and 2016. Although it is possible additional tests were performed but not reported to SALVH, this finding raises doubt concerning the representativeness of this sample population of PLHIV on ART. Further, records for a significant proportion of individuals (46.3%) who met the study criteria (i.e. ART initiation between 2013 and 2016 followed by a viral load test within 3–12 months after) had unknown virologic status in the national surveillance system. This led us to exclude these individuals from the primary analysis and called into question potential selection bias. Our bias analysis indicated that individuals included in the study were more likely to be treated with 3TC-EFV-TDF and have a CD4 count below 200 cells/mm^3^. While having a low CD4 count was associated with increased risk of delayed viral suppression in the primary analysis, treatment with 3TC-EFV-TDF was associated with reduced risk, and therefore these differences were not systematically associated with the outcome. Another limitation of the current analysis was the lack of a baseline viral load test and the use of only a single viral load measurement to classify viral suppression. Ideally, two viral load test results would be used to confirm a diagnosis of viral failure, however, baseline and/or follow-up testing was not sufficiently available in this sample to be included in the analysis. Additionally, per our inclusion criteria of viral load assessment between 3 and 12 months of ART initiation, we found fewer samples tested in the 3-6-month window. Despite this, findings from the viral load boxplot demonstrated consistent viral load values across all months of assessment. Therefore, it is unlikely that assessment within the 3-6-month window led to a biased assessment of delayed viral suppression in this analysis.

The HIV treatment guidelines in Haiti, as of 2018, recommend performing a second viral load test six months after the initial test for individuals with an initial viral load ≥1,000 copies/mL to confirm a diagnosis of virologic failure. However, because multiple assessments were not sufficiently available for consideration in this analysis, we opted to examine delayed viral suppression instead. Further, the proportion of missing data on some covariates ranged from 14.9% to 35.9% which limited their utility in the analyses. Of note, information on the history or presence of TB or STIs was significantly under-reported (33.0% and 28.3% missing, respectively). This may explain the lack of an association between virologic outcomes and the history of or comorbid tuberculosis, the latter of which was previously identified as a factor for delayed viral suppression in this population [6]. Additionally, WHO clinical stage data was missing for approximately 35.9% of individuals, which likely explains the lack of association between delayed viral suppression and more severe infection stages. Further, limited or no data were available in SALVH on the mode of HIV transmission, treatment adherence or prior treatment exposure, which have previously been linked to delays in viral suppression.

## Conclusions

In conclusion, this study was the first to assess factors associated with delayed viral suppression among PLHIV initiating first-line ART outside of Port-au-Prince, Haiti using the national centralized surveillance system. As a result, we identified opportunities for public health action to inform national treatment guidelines and improve patient care. Virologic status differed by various demographic and clinical factors, including age, clinic department, ART regimen, CD4 count, and receipt of ART from multiple treatment facilities. The alternative ART regimen 3TC-AZT-NVP was strongly associated with poorer virologic outcomes in the present study, and therefore we recommend more frequent viral load testing for individuals receiving these first-line therapies. Strategies to improve ART outcomes, such as more frequent viral load testing, drug resistance testing, and ART adherence counseling, are recommended for all PLHIV with delayed suppression at the first viral load assessment. Additionally, given that missingness was associated with poorer health outcomes in this analysis, sustained efforts to maintain clinical data flows into SALVH are needed. Furthermore, future studies should investigate the underlying socio-ecological, behavioral and/or healthcare system factors contributing to poorer ART outcomes by clinic department.

## Supporting information

S1 Table Demographic and clinical characteristics of persons living with HIV in Haiti by viral load test status, 2013–2017. (DOCX)

S1 Fig Distribution of viral load values by month of assessment for PLHIV with delayed viral suppression. The figure displays the mean, quartiles, minimum, and maximum log-transformed viral load values by the month of viral load test following ART initiation. These results indicate stable viral load values across each month of assessment.
(PNG)

## References

[1] World Health Organization. Consolidated Guidelines on the Use of Antiretroviral Drugs for Treating and Preventing HIV Infection: Recommendations for a Public Health Approach. Geneva; 2013 Available: http://www.ncbi.nlm.nih.gov/books/NBK195400/24716260

[2] Lynen L, Van Griensven J, Elliott J. Monitoring for treatment failure in patients on first-line antiretroviral treatment in resource-constrained settings. Curr Opin HIV AIDS. 2010;5: 1–5. 10.1097/COH.0b013e3283333762 20046141

[3] Chun HM, Obeng-Aduasare YF, Broyles LN, Ellenberger D. Expansion of Viral Load Testing and the Potential Impact on Human Immunodeficiency Virus Drug Resistance. The Journal of Infectious Diseases. 2017;216: S808–S811. 10.1093/infdis/jix404 29029178 PMC5745574

[4] McNairy ML, Joseph P, Unterbrink M, Galbaud S, Mathon J-E, Rivera V, et al Outcomes after antiretroviral therapy during the expansion of HIV services in Haiti. Vermund SH, editor. PLOS ONE. 2017;12: e0175521 10.1371/journal.pone.0175521 28437477 PMC5402937

[5] Auld AF, Pelletier V, Robin E, Shiraishi RW, Dee J, Antoine M, et al Retention throughout the HIV Care and Treatment Cascade: from Diagnosis to Antiretroviral Treatment of Adults and Children Living with HIV–Haiti, 1985–2015. Morbidity and Mortality Weekly (MMWR). 10.4269/ajtmh.17-0116 PMC5676635 29064357

[6] Jean Louis F, Buteau J, François K, Hulland E, Domerçant JW, Yang C, et al Virologic outcome among patients receiving antiretroviral therapy at five hospitals in Haiti. PLoS ONE. 2018;13: e0192077 10.1371/journal.pone.0192077 29381736 PMC5790273

[7] Charles M, Noel F, Leger P, Severe P, Riviere C, Beauharnais CA, et al Survival, plasma HIV-1 RNA concentrations and drug resistance in HIV-1-infected Haitian adolescents and young adults on antiretrovirals. Bull World Health Organ. 2008;86: 970–977. 10.2471/blt.07.050120 19142298 PMC2649577

[8] Cesar C, Jenkins CA, Shepherd BE, Padgett D, Mejía F, Ribeiro SR, et al Incidence of virological failure and major regimen change of initial combination antiretroviral therapy in the Latin America and the Caribbean: an observational cohort study. Lancet HIV. 2015;2: e492–500. 10.1016/S2352-3018(15)00183-6 26520929 PMC4651009

[9] Hare AQ, Ordóñez CE, Johnson BA, del Rio C, Kearns RA, Wu B, et al Gender-Specific Risk Factors for Virologic Failure in KwaZulu-Natal: Automobile Ownership and Financial Insecurity. AIDS and Behavior. 2014;18: 2219–2229. 10.1007/s10461-014-0849-1 25037488 PMC4198463

[10] Delcher C, Puttkammer N, Arnoux R, Francois K, Griswold M, Zaidi I, et al Validating Procedures used to Identify Duplicate Reports in Haiti’s National HIV/AIDS Case Surveillance System. J Registry Manag. 2016;43: 10–15. 27195993 PMC5222994

[11] Smit PW, Sollis KA, Fiscus S, Ford N, Vitoria M, Essajee S, et al Systematic Review of the Use of Dried Blood Spots for Monitoring HIV Viral Load and for Early Infant Diagnosis. Paxton WA, editor. PLoS ONE. 2014;9: e86461 10.1371/journal.pone.0086461 24603442 PMC3945725

[12] Pannus P, Claus M, Gonzalez MMP, Ford N, Fransen K. Sensitivity and specificity of dried blood spots for HIV-1 viral load quantification: A laboratory assessment of 3 commercial assays. Medicine. 2016;95: e5475 10.1097/MD.0000000000005475 27902602 PMC5134769

[13] World Health Organization. ANNEX 10, WHO clinical staging of HIV disease in adults, adolescents and children. Geneva, Switzerland; 2016. Available: https://www.ncbi.nlm.nih.gov/books/NBK374293/

[14] SAS Institute. SAS/STAT 9.4 User’s Guide. Cary, NC: SAS Institute, Inc; 2015.

[15] ESRI. ArcGIS Pro 2.3. Redlands, CA: Environmental Systems Research Institute; 2019.

[16] Jean SS, Pape JW, Verdier RI, Reed GW, Hutto C, Johnson WD, et al The natural history of human immunodeficiency virus 1 infection in Haitian infants. Pediatr Infect Dis J. 1999;18: 58–63. 10.1097/00006454-199901000-00014 9951982

[17] Haile-Selassie H, World Health Organization, World Health Organization, Department of HIV/AIDS, Centers for Disease Control and Prevention (U.S.), Global Fund to Fight AIDS T and Malaria, et al WHO HIV drug resistance report 2017. 2017 Available: http://apps.who.int/iris/bitstream/10665/255896/1/9789241512831-eng.pdf

[18] Rupérez M, Pou C, Maculuve S, Cedeño S, Luis L, Rodríguez J, et al Determinants of virological failure and antiretroviral drug resistance in Mozambique. J Antimicrob Chemother. 2015;70: 2639–2647. 10.1093/jac/dkv143 26084302

[19] Ayele T, Jarso H, Mamo G. Immunological outcomes of Tenofovir versus Zidovudine-based regimens among people living with HIV/AIDS: a two years retrospective cohort study. AIDS Research and Therapy. 2017;14 10.1186/s12981-017-0141-3 28143541 PMC5286788

[20] Velen K, Lewis JJ, Charalambous S, Grant AD, Churchyard GJ, Hoffmann CJ. Comparison of Tenofovir, Zidovudine, or Stavudine as Part of First-Line Antiretroviral Therapy in a Resource-Limited-Setting: A Cohort Study. Le Grand R, editor. PLoS ONE. 2013;8: e64459 10.1371/journal.pone.0064459 23691224 PMC3653880

[21] Mbuagbaw L, Mursleen S, Irlam JH, Spaulding AB, Rutherford GW, Siegfried N. Efavirenz or nevirapine in three-drug combination therapy with two nucleoside or nucleotide-reverse transcriptase inhibitors for initial treatment of HIV infection in antiretroviral-naïve individuals. Cochrane Database Syst Rev. 2016;12: CD004246 10.1002/14651858.CD004246.pub4 27943261 PMC5450880

[22] Alvarez-Uria G, Naik PK, Pakam R, Midde M. Early HIV viral load determination after initiating first-line antiretroviral therapy for indentifying patients with high risk of developing virological failure: data from a cohort study in a resource-limited setting. Trop Med Int Health. 2012;17: 1152–1155. 10.1111/j.1365-3156.2012.02982.x 22487689

[23] Hermans LE, Moorhouse M, Carmona S, Grobbee DE, Hofstra LM, Richman DD, et al Effect of HIV-1 low-level viraemia during antiretroviral therapy on treatment outcomes in WHO-guided South African treatment programmes: a multicentre cohort study. The Lancet Infectious Diseases. 2018;18: 188–197. 10.1016/S1473-3099(17)30681-3 29158101

[24] Kerschberger B, Boulle AM, Kranzer K, Hilderbrand K, Schomaker M, Coetzee D, et al Superior virologic and treatment outcomes when viral load is measured at 3 months compared to 6 months on antiretroviral therapy. Journal of the International AIDS Society. 2015;18: 20092 10.7448/IAS.18.1.20092 26403636 PMC4582072

[25] Charles M, Leger PD, Severe P, Guiteau C, Apollon A, Gulick RM, et al Virologic, clinical and immunologic responses following failure of first-line antiretroviral therapy in Haiti. J Int AIDS Soc. 2012;15: 17375 10.7448/IAS.15.2.17375 22713258 PMC3499802

