# Supplementary material for: Risk factors for delayed viral suppression on first-line antiretroviral therapy among persons living with HIV in Haiti, 2013–2017
Source: PLoS One. 2020 Oct 29;15(10):e0240817. doi: 10.1371/journal.pone.0240817 (PMC7595392; doi:10.1371/journal.pone.0240817)
Supplement: S1 Table — (DOCX) [file pone.0240817.s001.docx]

**S1 Table**. Demographic and clinical characteristics of persons living with HIV in Haiti by viral load test status, 2013-2017.

| Characteristic | | Known viral status (n=3,368) | Unknown viral status (n=3,027) | Global p-for difference χ² test |
| --- | --- | --- | --- | --- |
| Mean (SD) | | | | |
| Age at HIV diagnosis (years)^*^ | | 35.1 (13.5) | 34.7 (13.4) | 0.2652 |
| Frequency (%) | | | | |
| Age at HIV diagnosis (years) | |  |  | 0.3970 |
| <5 | | 98 (2.9) | 88 (2.9) |  |
| 5-14 | | 95 (2.8) | 103 (3.4) |  |
| 15-19 | | 101 (3.0) | 80 (2.6) |  |
| 20-29 | | 813 (24.1) | 703 (23.2) |  |
| 30-39 | | 1,034 (30.7) | 960 (31.7) |  |
| 40-49 | | 687 (20.4) | 605 (20.0) |  |
| 50-59 | | 350 (10.4) | 310 (10.2) |  |
| 60+ | | 119 (3.5) | 92 (3.0) |  |
| Unknown | | 71 (2.1) | 86 (2.8) |  |
| Sex | |  |  | 0.0025 |
| Female | | 1,989 (59.1) | 1,887 (62.3) |  |
| Male | | 1,379 (40.9) | 1,136 (37.5) |  |
| Unknown | | - | 4 (0.1) |  |
| Marital status | |  |  | 0.0063 |
| Single | | 652 (19.4) | 569 (18.8) |  |
| Cohabitating | | 1,294 (38.4) | 1,131 (37.4) |  |
| Married | | 481 (14.3) | 404 (13.3) |  |
| Divorced or widowed | | 439 (13.0) | 367 (12.1) |  |
| Unknown | | 502 (14.9) | 556 (18.4) |  |
| Clinic department^†^ | |  |  | <0.0001 |
| Artibonite-Centre | | 279 (8.3) | 215 (7.1) |  |
| Nord | | 642 (19.1) | 573 (18.9) |  |
| Nord-Est | | 160 (4.8) | 72 (2.4) |  |
| Nord-Ouest | | 82 (2.4) | 138 (4.6) |  |
| Ouest | | 1,919 (57.0) | 1,759 (58.1) |  |
| Sud | | 278 (8.3) | 265 (8.8) |  |
| Unknown | | 8 (0.2) | 5 (0.2) |  |
| Year of HIV diagnosis |  | |  | <0.0001 |
| <2010 | 195 (5.8) | | 277 (9.2) |  |
| 2010-11 | 200 (5.9) | | 191 (6.3) |  |
| 2012-13 | 518 (15.4) | | 489 (16.2) |  |
| 2014-15 | 1,474 (43.8) | | 1,095 (36.2) |  |
| 2016-17 | 969 (28.8) | | 959 (31.7) |  |
| Unknown | 12 (0.4) | | 16 (0.5) |  |
| WHO clinical stage |  | |  | <0.0001 |
| I | 386 (11.5) | | 341 (11.3) |  |
| II | 809 (24.0) | | 596 (19.7) |  |
| III | 364 (10.8) | | 298 (9.8) |  |
| IV | 58 (1.7) | | 57 (1.9) |  |
| Unknown | 1,751 (52.0) | | 1,735 (57.3) |  |

**S1 Table**. Continued.

| Characteristic | Known viral status (n=3,368) | Unknown viral status (n=3,027) | Global p-for difference χ² test |
| --- | --- | --- | --- |
| Frequency (%) |  |  |  |
| Year of ART initiation |  |  | <0.0001 |
| 2013 | 230 (6.8) | 176 (5.8) |  |
| 2014 | 261 (7.7) | 294 (9.7) |  |
| 2015 | 1,026 (30.5) | 555 (18.3) |  |
| 2016 | 1,851 (55.0) | 2,002 (66.1) |  |
| ART regimen |  |  | 0.0014 |
| 3TC-AZT-NVP | 133 (3.9) | 145 (4.8) |  |
| 3TC-EFV-AZT | 130 (3.9) | 172 (5.7) |  |
| 3TC-EFV-TDF | 2,959 (87.9) | 2,557 (84.5) |  |
| Other | 146 (4.3) | 153 (5.1) |  |
| Earliest CD4 T cell count |  |  | <0.0001 |
| <200 | 810 (24.0) | 456 (15.1) |  |
| 200-499 | 1,049 (31.1) | 829 (27.4) |  |
| 500+ | 840 (24.9) | 853 (28.2) |  |
| Unknown | 669 (19.9) | 889 (29.4) |  |
| History/presence of TB |  |  | <0.0001 |
| No | 2,102 (62.4) | 1,715 (56.7) |  |
| Yes | 153 (4.5) | 94 (3.1) |  |
| Unknown | 1,113 (33.0) | 1,218 (40.2) |  |
| History/presence of STIs |  |  | <0.0001 |
| No | 1,653 (49.1) | 1,417 (46.8) |  |
| Yes | 760 (22.6) | 539 (17.8) |  |
| Unknown | 955 (28.4) | 1,071 (35.4) |  |
| Any risk factor identified |  |  | <0.0001 |
| No | 1,549 (46.0) | 1,664 (55.0) |  |
| Yes | 1,819 (54.0) | 1,363 (45.0) |  |
| Multiple provider episodes |  |  | 0.9752 |
| No | 3,215 (95.5) | 2,889 (95.4) |  |
| Yes | 153 (4.5) | 138 (4.6) |  |
| Time to ART |  |  | <0.0001 |
| < 1 year of diagnosis | 2,354 (69.9) | 1,845 (61.0) |  |
| ≥ 1 year of diagnosis | 952 (28.3) | 1,118 (36.9) |  |
| Unknown | 62 (1.8) | 64 (2.1) |  |

SD, standard deviation; WHO, World Health Organization; ART, antiretroviral therapy; 3TC, lamivudine; AZT, zidovudine; NVP, nevirapine; EFV, efavirenz; TDF, tenofovir; TB, tuberculosis; STIs, sexually transmitted infections

*Evaluated using t-test. †Sud department is combined and includes Sud, Sud-Est, Grande-Anse, Nippes.
