# Supplementary figures and images for: Risk factors for delayed viral suppression on first-line antiretroviral therapy among persons living with HIV in Haiti, 2013–2017
Source: PLoS One. 2020 Oct 29;15(10):e0240817. doi: 10.1371/journal.pone.0240817 (PMC7595392; doi:10.1371/journal.pone.0240817)

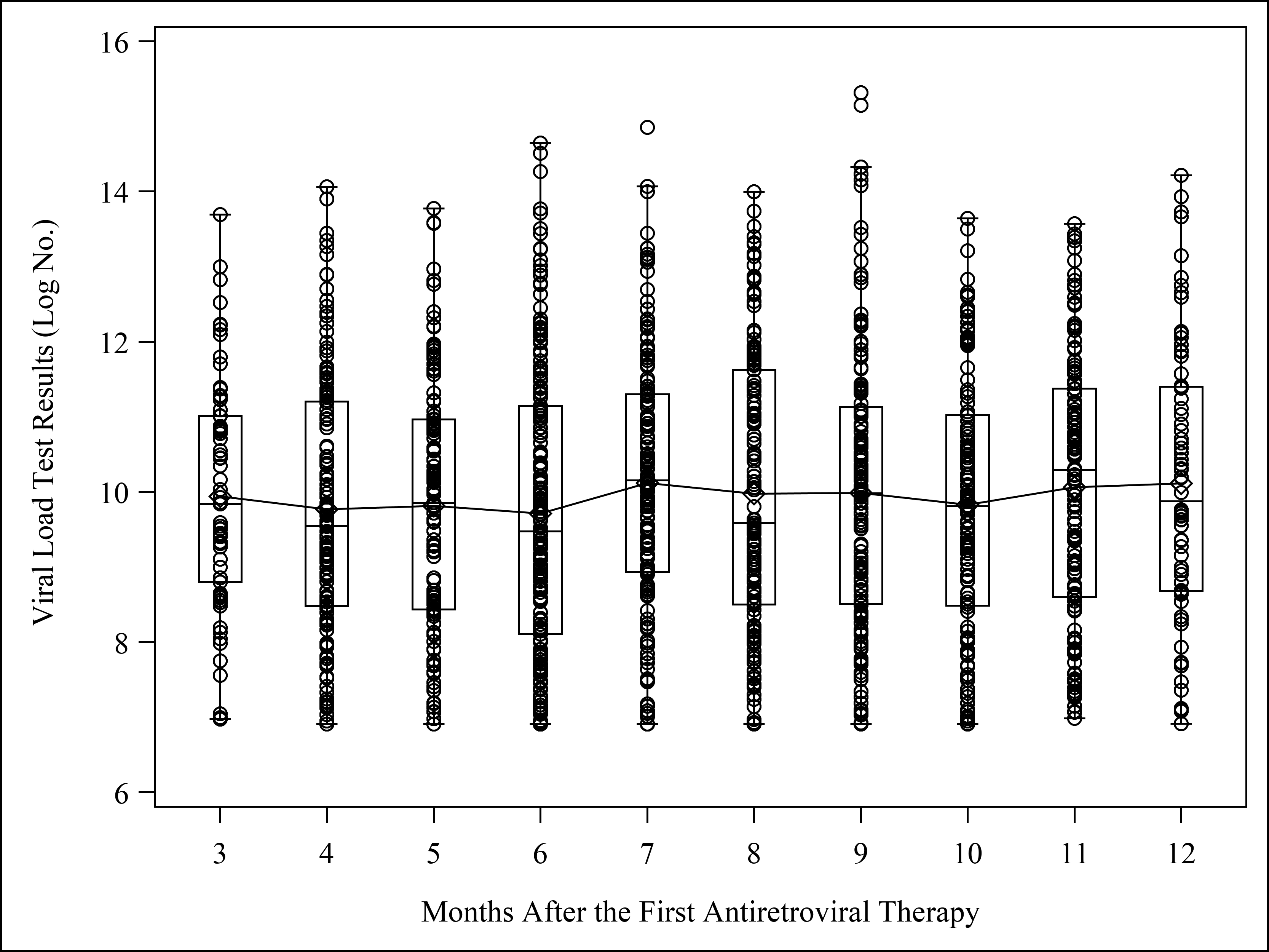

Supplement: S1 Fig — The figure displays the mean, quartiles, minimum, and maximum log-transformed viral load values by the month of viral load test following ART initiation. These results indicate stable viral load values across each month of assessment. (PNG) [file pone.0240817.s002.png]
